# Assessment of Knowledge, Attitudes, and Practices Towards Monkeypox and Associated Factors Among Undergraduate Healthcare Students in Ethiopia, 2025: A Cross‐Sectional Study

**DOI:** 10.1002/hsr2.72513

**Published:** 2026-05-11

**Authors:** Esubalew Muluneh Aligaz, Zekarias Markos, Fikadu Tadesse Diress, Sitotaw Tesfa Zegeye

**Affiliations:** ^1^ Department of Anesthesia Bahir Dar University College of Medicine and Health Science Bahir Dar Ethiopia; ^2^ Department of Anesthesia Wachamo University College of Medicine and Health Science Hossana Ethiopia

**Keywords:** emerging infectious diseases, Ethiopia, knowledge‐attitude‐practice, Mpox, University students

## Abstract

**Background:**

Mpox has emerged as a re‐emerging zoonotic disease of global public health concern, particularly affecting regions with limited awareness and preparedness. University students represent a critical population for disease prevention due to their mobility, social interaction, and role as future professionals.

**Aims:**

To assess the knowledge, attitudes, and practices regarding Monkeypox virus among undergraduate students in Central Ethiopia.

**Methods:**

An institution‐based cross‐sectional study was conducted among 410 undergraduate healthcare students. Data were collected using a structured, self‐administered questionnaire covering socio‐demographic characteristics and knowledge, attitude, and practice related to Mpox. Knowledge, attitude, and practice levels were categorized using Bloom's cut‐off points. Logistic regression analyses were performed to identify factors associated with knowledge, attitudes, and practices, with statistical significance set at *p* < 0.05.

**Results:**

Among 410 medical and health science graduates, 85% had heard of Mpox. While most correctly identified it as a viral disease (92.6%) and knew its major transmission routes (85%), knowledge gaps existed regarding vaccination (26.8%) and incubation period (16%), and 41.4% incorrectly cited antibiotics as treatment. Overall, 43.9% had good knowledge, 7.3% moderate, and 48.7% poor. A positive attitude was reported by 75.6%, with great concern about disease spread (80.4%) and willingness to self‐isolate (83.1%), though only 29.2% trusted the healthcare system's preparedness. Preventive practices were generally good (76.1%), including frequent hand hygiene (85.3%) and mask use (90.2%). Prior awareness, adequate knowledge, and a positive attitude were significantly linked to better preventive practices.

**Conclusion:**

Despite favorable attitudes and practices, undergraduate healthcare students exhibited important knowledge gaps regarding Mpox. Strengthening curriculum integration, targeted training, and access to reliable information sources is essential to improve preparedness for emerging infectious diseases.

**Abbreviations**
CGPA: cumulative grade point average
HCP: healthcare professionals
KAP: knowledge, attitude, and practice
Mpox: Monkeypox
WHO: World Health Organization
W/U/N/E/M/M/C/H: Wachamo University Nigist Eleni Mohammed Memorial Comprehensive Hospital

## 1 Introduction

Emerging and re‐emerging infectious diseases continue to pose significant challenges to global health systems, particularly in low‐ and middle‐income countries where surveillance, awareness, and preparedness may be limited. Monkeypox (Mpox), a zoonotic viral disease caused by the monkeypox virus, has gained renewed international attention due to its expanding geographic distribution and outbreak potential beyond traditionally endemic regions.

Mpox is a zoonotic viral disease caused by the mpox virus, a member of the Orthopoxvirus genus within the Poxviridae family. It is closely related to the smallpox virus and is characterized clinically by a pox‐like rash accompanied by systemic symptoms. Historically, the disease has been concentrated in Central and West Africa, with transmission primarily occurring from infected animals, particularly rodents, to humans. However, human‐to‐human transmission can also occur through close physical contact, respiratory droplets, or exposure to contaminated materials [1]. In recent years, cases have been reported outside endemic areas, including in the United Kingdom and the United States, signaling a shift in the epidemiological pattern of the disease [2]

Since May 2022, a multicountry outbreak has significantly altered the global health landscape, prompting the World Health Organization (WHO) to declare mpox a Public Health Emergency of International Concern and urging countries to implement rapid surveillance and control [3, 4].

Transmission occurs through direct contact with infected animals or human lesions, respiratory secretions during prolonged close contact, or contaminated objects. Common clinical manifestations include fever, headache, lymphadenopathy, chills, fatigue, and a characteristic vesiculopustular rash that typically progresses from the face to other parts of the body [5]. While many cases are self‐limiting, the disease can occasionally result in severe complications and death. Although there is no widely available specific antiviral therapy, supportive care remains the mainstay of treatment, and prior smallpox vaccination offers partial cross‐protection [6]. Preventive strategies rely heavily on infection‐control practices, hygiene measures, and avoidance of exposure to infected individuals or animals.

Education and awareness are critical components of outbreak prevention and control. Targeted health education programs, particularly among medical and health science students, can play a vital role in improving understanding of transmission dynamics, risk perception, and preventive behaviors [7]. University healthcare students represent a unique and important population: they are at increased risk of exposure during training, are actively engaged in clinical or community environments, and often serve as trusted sources of information within their families and communities [8, 9].

Moreover, university students are socially active, highly mobile, and deeply connected through digital and community networks, factors that can both increase transmission risk and provide opportunities for rapid dissemination of accurate health information. As future healthcare professionals and community leaders, their knowledge and attitudes toward emerging infections are essential for strengthening long‐term public health resilience.

Despite growing global concern, relatively few studies have assessed healthcare students' knowledge, attitudes, and practices regarding mpox [10, 11]. Existing evidence suggests that significant knowledge gaps and misconceptions persist among students, which may hinder effective prevention and response efforts. In Ethiopia, where resources for emerging disease preparedness remain constrained, there is particularly limited evidence regarding awareness and behavioral readiness related to mpox among young adults [12].

University students constitute an important population in infectious disease prevention efforts. Their high level of social interaction, mobility, and engagement in communal environments increases the risk of disease transmission. Moreover, students serve as future professionals and community leaders, making their understanding of emerging diseases crucial for strengthening long‐term public health resilience [11].

Despite increasing global concern, there is limited evidence regarding awareness and behavioral preparedness related to Mpox among young adults in Ethiopia. Assessing knowledge, attitudes, and practices is essential to identify misconceptions, guide health education interventions, and support national preparedness strategies.

Therefore, this study aimed to assess the knowledge, attitudes, and practices regarding Mpox among undergraduate students in Central Ethiopia.

## 2 Methods

### 2.1 Study Design and Setting

An institution‐based cross‐sectional study was conducted from 10 May to 30 June 2025 among undergraduate healthcare students at Wachamo University and Nigist Eleni Mohammed Memorial Comprehensive Specialized Hospital, located in Central Ethiopia. The study aimed to assess students' knowledge, attitudes, and practices (KAP) toward mpox and identify associated factors.

### 2.2 Study Population

The source population included all undergraduate students enrolled in Medicine, Nursing, Midwifery, Medical Laboratory Sciences, and Anesthesia programs who were actively involved in clinical training during the study period.

### 2.3 Eligibility Criteria

Undergraduate healthcare students enrolled in health‐related programs during the study period were considered eligible to participate. The study included registered students who were present at the time of data collection and actively engaged in clinical attachments or hospital‐based training. Participation was voluntary, and only those who were willing to take part and provided informed consent were included.

Students who were absent during the data collection period were excluded. In addition, participants who were involved in the pretest phase, as well as those who missed or did not complete the questionnaire, were excluded from the final analysis.

### 2.4 Sample Size Determination and Sample Size Technique

The study population comprised all undergraduate healthcare students, including medicine, medical laboratory technology, nursing, midwifery, and anesthesia, who were undergoing training at Wachamo University and practicing at Nigist Eleni Mohammed Memorial Comprehensive Hospital during the study period.

The sample size was calculated using a single population proportion formula, as no previous study had assessed the knowledge, attitude, and practice (KAP) toward the Mpox virus among undergraduate healthcare students in the study area. Therefore, a prevalence (p) of 50% was assumed to obtain the maximum sample size.

### 2.5 Formula Used

n=(Zα/2)²×p(1−p)/d²

Where:

- *n* = minimum required sample size
- Zα/2 = standard normal value at 95% confidence level (1.96)
- *p* = assumed prevalence of KAP toward Mpox (0.5)
- *d* = margin of error (0.05)

Substituting these values into the formula yielded the minimum sample size. After adding a 10% allowance for potential non‐response, the final sample size was 422 participants.

A stratified sampling technique was employed to classify graduate class students according to their professional field (medicine, nursing, midwifery, anesthesia, and medical laboratory technology). The total sample size (*N* = 410) was proportionally allocated to each stratum based on the number of eligible students in each discipline. The proportional allocation was calculated using the formula:

$$n_{i}{=(N}_{i}/N)\times n,$$

where *nᵢ* represents the sample size for each stratum, *Nᵢ* is the total number of eligible students in each department, *N* is the total number of eligible students across all departments, and *n* is the final sample size.

**Figure 1:**
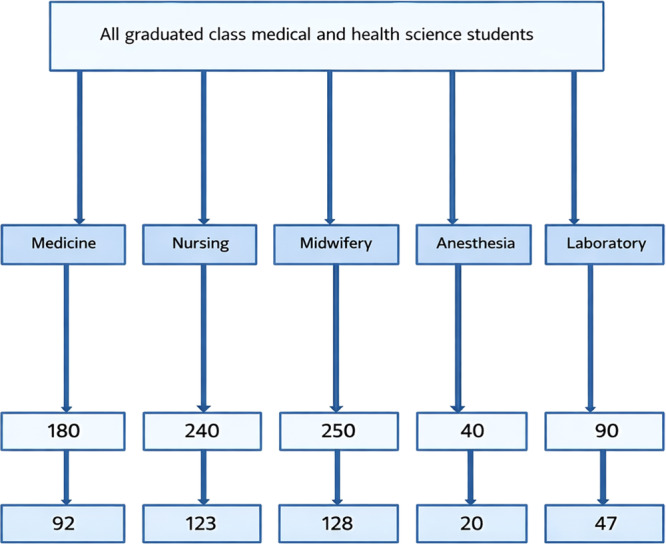
Flowchart showing proportional allocation of the study sample among graduated medical and health science students by department.

Within each stratum, participants were selected using a systematic random sampling technique. The sampling interval (*k*) was determined by dividing the total number of students in each stratum by the allocated sample size for that stratum (*k* = *Nᵢ*/*nᵢ*). The first participant was selected using a simple random sampling method (lottery method), and subsequent participants were selected at every kth interval until the required sample size was obtained. This procedure ensured proportional and unbiased representation of all professional groups (Figure 1).

### 2.6 Data Collection and Procedure

Data were collected electronically using the KoboCollect mobile application to enhance data accuracy, minimize entry errors, and facilitate real‐time data management [13]. A total of three trained data collectors were responsible for recruiting participants, explaining the objectives of the study, and guiding students through the electronic questionnaire submission process. One supervisor closely monitored the overall data collection process, ensuring completeness, consistency, and daily synchronization of submitted data.

All data collectors and the supervisor received 2 days of training before the commencement of data collection. The training focused on the study protocol, ethical considerations, use of the KoboCollect application, and methods for ensuring data quality. Data were obtained using a self‐administered, standardized questionnaire adapted from previously published studies [14, 15, 16, 17]. The questionnaire consisted of several sections. The first section collected socio‐demographic information, including age, sex, marital status, last cumulative grade point average (CGPA), and sources of information about Mpox. The subsequent sections assessed participants' knowledge, attitudes, and practices regarding Mpox infection. The full questionnaire is provided as Supporting Material.

Knowledge of Mpox was evaluated using 11 structured items covering symptoms, transmission, prevention, treatment, epidemiology, and immunization. Each knowledge question had three response options: “Yes,” “No,” and “I don't know.” Attitudes toward Mpox were measured using a three‐point Likert scale (agree, neutral, disagree), assessing perceptions of disease severity, willingness to accept isolation if infected, confidence in preventive measures, and beliefs regarding the ability to control and manage potential outbreaks.

### 2.7 Section One: Socio‐Demographic Data

This section consisted of 8 questions aimed at gathering demographic information about the participants, including age, gender, marital status, profession, last CGPA source of information, and information heard before.

### 2.8 Section Two: Knowledge Assessment

Comprising 11 questions, this section focuses on evaluating the knowledge of healthcare undergraduate students regarding mpox. The questions covered various aspects of the disease, such as its transmission, symptoms, and treatment options. The questions were phrased using a combination of yes/no. Each correct answer was assigned a score of “1,” while incorrect answers received a score of “0.” Participants' knowledge was categorized as Good (≥ 70% of total score), Fair (50%–69% of total score), or Poor (< 50% of total score) based on their overall score [12, 18, 19].

### 2.9 Section Three: Attitude Assessment

This section included eight questions rated on a 3‐point Likert scale. It aimed to explore the attitudes of healthcare professionals towards mpox. Attitudes were categorized as Positive (≥ 70% of total score), Moderate (50%–69% of total score), or Negative (< 50% of total score) based on the total score accumulated from the responses [12, 18, 19].

### 2.10 Section Four: Practice Assessments

This section includes seven items that investigate self‐care and prevention practices towards Mpox. A yes or no response was given to each question. Scores of 1 (yes) and 0 (no) were given to the right and wrong practices, respectively. were categorized as good practice (≥ 70% of total score) and poor practice(< 50%of total score) [17].

### 2.11 Data Quality Control

Several measures were implemented to ensure the completeness, accuracy, and reliability of the collected data. Daily supervision and on‐site verification were conducted throughout the data collection period. In addition, automated validation checks embedded within the KoboCollect system were used to minimize missing values and reduce data entry inconsistencies.

A pretest was conducted on 5% of the total sample size before the actual data collection to assess the clarity, relevance, and feasibility of the questionnaire. Based on the findings of the pretest, necessary modifications were made to improve the instrument. The internal consistency of the tool was evaluated using Cronbach's alpha, which yielded a reliability coefficient of 0.77, indicating acceptable reliability.

### 2.12 Statistical Analysis

The collected data was checked for its completeness, and then, the data was imported into SPSS version 26. The data was summarized by statistical analysis for descriptive statistics of the variables. For categorical variables, frequencies and percentages were calculated. The bivariate and multivariate logistic regression model was used for the assessment of associated factors linked to the independent variables and the results. We computed odds ratios (OR) with 95% confidence intervals (95% CI). The multivariate logistic regression model for associated factor analysis incorporated all variables with a *p* ≤ 0.25 [20]. Regression diagnostic checks were conducted before the multivariable analysis to ensure the validity of the model. Multicollinearity was assessed using the Variance Inflation Factor (VIF), and all variables showed acceptable values (VIF < 10). Model fitness was evaluated using the Hosmer–Lemeshow goodness‐of‐fit test, which indicated an adequate fit (*p* > 0.05). All statistical tests were two‐sided, and a *p*‐value of < 0.05 was considered statistically significant.

## 3 Results

### 3.1 Socio‐Demographic Characteristics of Medical and Health Science Students

A total of 410 undergraduate healthcare students participated in the study. The majority were aged 21–22 years (64.4%), followed by 18–20 years (33.4%), while only 2.2% were aged 23–25 years. Females constituted 58% of the respondents, whereas males accounted for 42%. Most participants were single (92.5%).

Regarding the field of study, midwifery students represented the largest group (31%), followed by nursing (30%), medicine (23%), laboratory sciences (11%), and anesthesia (5%). The majority of students had a CGPA between 2.5 and 3.74 (82.4%), while 16.1% had a CGPA ≥ 3.75.

Most respondents (85%) reported that they had previously heard about Mpox. The main sources of information were websites of hospitals/Ministry of Health (37.6%) and social media (26.8%), followed by friends/family (15.9%), television (14.6%), and formal training (7.6%) (Table 1).

**Table 1:** Socio‐demographic characteristics of participants (*n* = 410).

Variables	Categories	Frequency (*n*)	Percentage (%)
Age (years)	18–20	137	33.4
	21–22	264	64.4
	23–25	9	2.2
Sex	Male	172	42
	Female	238	58
Marital status	Single	380	92.5
	Married	30	7.5
Field of study	Medicine	92	23
	Nursing	123	30
	Midwifery	128	31
	Anesthesia	20	5
	Laboratory	47	11
Last CGPA	< 2.5	6	1.5
	2.5–3.74	338	82.4
	≥ 3.75	66	16.1
Heard Mpox previously	Yes	350	85
	No	60	15
Primary information source	Television/Mass media	60	14.6
	Social media	110	26.8
	Health professionals	7	1.7
	Websites of the hospital/Ministry of Health	154	37.6
	Friends/Family	65	15.9
	Training	31	7.6

### 3.2 Knowledge of Medical and Health Science Students Towards Mpox

Most participants demonstrated a good understanding of the basic etiology and transmission of Mpox. A large proportion correctly identified the disease as being caused by a virus (92.6%) and recognized key transmission routes, including contact with infected persons or animals (70%) and exposure to lesions, body fluids, or respiratory droplets (85%). Awareness of common clinical manifestations was also relatively high, with 73.3% identifying rash and 60.9% recognizing fever as symptoms.

Despite these strengths, notable knowledge gaps were observed in more specific areas. Only 26.8% of students were aware that a vaccine is available for Mpox, and just 19.5% recognized the cross‐protective role of the smallpox vaccine. Furthermore, only 16% correctly identified the typical incubation period, and a considerable proportion (41.4%) mistakenly believed that antibiotics are used for treatment, indicating persistent misconceptions regarding disease management.

Using Bloom's cut‐off classification, 43.9% of participants were categorized as having good knowledge (≥ 70%), while 7.3% demonstrated moderate knowledge (50%–69%). Nearly half of the respondents (48.7%) fell into the poor knowledge category (< 50% (Table 2).

**Table 2:** Knowledge of Mpox among undergraduate healthcare students (*n* = 410).

Statement	Response	Correct response *n*	Perecentage
Knowledge statement	The causative agent for mpox infection is a virus	380	92.6
	Mpox can be transmitted through a bite from an infected Mpox	310	75
	Travelers from America and Europe are the primary source of imported cases of Mpox	220	53
	There is no specific treatment for Mpox	100	24.3
	People who got the chickenpox vaccine are immunized against Mpox	80	19.5
	Mpox is more prevalent in Western and Central Africa	200	48.7
	Antibiotics are used to treat human Mpox	170	41.4
	Pregnant women are at increased risk for severe Mpox infection	180	43.9
	The average incubation period of Mpox infection is 7‐14 days	66	16
	The elderly group is at a higher risk of getting Mpox infection	140	34.1
	A smallpox vaccine can be used for Mpox	80	19.5
	There is a vaccine against Mpox	110	26.8
Knowledge of the transmission route	Contact with an infected person or animal	290	70
	Contact with body lesions, body fluids, and respiratory droplets	351	85
	Sexual intercourse	50	12.1
	Contaminated objects	30	7.3
Awareness of symptoms	Fever	250	60.9
	Rash and painful skin lesion	300	73.3
	Lymphadenopathy	211	51.2
Knowledge of preventive measure	Hand hygiene	315	76.8
	Avoiding close contact with infected individuals	305	74.3
	Wear a mask	205	50

Score		Frequency (%)	Remark
< 50		200 (48.7)	Poor knowledge
50–69		30 (7.3)	Moderate knowledge
≥ 70		180(43.9)	Good knowledge

### 3.3 Factors Associated With Medical and Health Science Students' Knowledge About Mpox

Bivariate and multivariate logistic regression analyses were conducted to assess the association between healthcare undergraduate students' knowledge of Mpox and independent variables. In the bivariate analysis, source of information and having heard of Mpox before were selected as variables with a *p* ≤ 0.25. for multivariate regression analysis. In multivariate regression, the source of information, and having heard of Mpox before, were significantly associated factors.

Healthcare undergraduate students having information through training 13.28 times (AOR = 13.28: 95% CI: 6.48–7.20) were more likely to have good knowledge than those who had information through television. Likewise, health care undergraduate students who had access to information about social media, through friends and Websites of the hospital/Ministry of Health, 5.57 times (AOR = 5.57: 95% CI: 2.54–12.23), 7.79 times (AOR = 7.96: 95% CI: 3.45–18.3), 8.86times (AOR = 8.63: 95% CI: 4.3–17.01) more likely to have good knowledge than those who access information through television, respectively. The odds of having good Mpox knowledge were 1.93 times (AOR = 1.93: 95% CI: 1.25–2.88) among healthcare students who didn't hear about Mpox, as shown in Table 3.

**Table 3:** Knowledge and associated factors Mpox among healthcare undergraduate students.

Variables	Knowledge level		COR (95% CI)	AOR (95% CI)	*p* value
	Good (%)	Poor (%)			
**Sex**					
Male	81 (19.7)	157 (38.2)	1		
Female	58 (14.1)	114 (27.8)	1.01 (0.67–1.53)		0.94
**Age in years**					
> 20	5 (1.2)	4 (0.9)	1		
22–22	89 (21.7)	175 (42.6)	2.55 (0.65–9.88)		1.77
22–25	45 (11.4)	92 (22.4)	2.45 (0.64–9.38)		1.88
**Field of study**					
Medi cine	32 (7.8)	60 (14.6)	1		
Nurse	39 (9.5)	84 (20.4)	3.84 (0.98–14.9)		0.52
Midwifery	46 (11.2)	82 (20)	1.21 (0.61–2.40)		0.58
Anesthetist	3 (0.7)	17 (4.1)	1.46 (0.72–2.93)		0.28
Laboratory	19 (4.6)	28 (6.8)	1.22 (0.61–2.62)	1.19 (0.26–1.45)	0.51
**Information source**					
Television	51 (12.4)	16 (3.9)	1	1	
Social media	20 (4.8)	35 (8.5)	5.01 (2.54–12.23)	5.57 (5.08–13.4)	**0.01**
Websites of the hospital/Ministry of Health	24 (5.8)	100 (24.3)	6.96 (3.45–18.3)	7.79 (4.03–11.1)	**0.01**
Friends/family	31 (7.5)	84 (20.4)	0.12 (0.06–0.25)	0.63 (0.3–2.01)	**0.01**
Training	14 (3.4)	35 (8.5)	5.18 (5.05–9.27)	13.28 (6.48–27.20)	**0.01**
**Last CGPA**					
< 2.5	7 (1.7)	9 (2.1)	1		
2.5–3.74	110 (26.8)	194 (47.3)	0.48 (0.16–1.42)		0.18
≥ 3.75	27 (6.5)	72 (17.5)	0.66 (0.40–1.10)		0.16
**Heard Mpox previously**					
Yes	86 (20.9)	123 (30)	1.93 (1.25–2.88)	1.74 (1.09–2.75)	**0.010**
No	54 (13.1)	147 (35.8)	1		

*Note: p* < 0.05 (bolded) indicates statistically significant associations.

Abbreviations: AOR, adjusted odds ratio; CI, confidence interval; COR, crude odds ratio.

### 3.4 Attitude Towards Mpox

Overall, students demonstrated a generally cautious and concerned attitude toward Mpox. The majority agreed that early detection can improve treatment outcomes (85.3%), believed that Mpox could impose an additional burden on healthcare systems (78%), and expressed concern about the potential spread of the disease within their communities (80.4%). A high proportion (83.1%) also reported willingness to self‐isolate if exposed, reflecting a strong sense of personal responsibility in preventing transmission.

However, confidence in the healthcare system's capacity to manage Mpox was relatively low, with only 29.2% expressing trust in its preparedness. In addition, just over half of the respondents (54.8%) indicated willingness to receive vaccination if it became available.

Based on the established scoring criteria, 75.6% of participants demonstrated a positive attitude (≥ 70%), while 7.4% had a fair attitude (50%–69%). A smaller proportion, 17%, were categorized as having a negative attitude (< 50%) (Table 4).

**Table 4:** Attitude towards Mpox (*n* = 410).

Statements	Response		
	A	*N*	D
I have bad feelings about the Mpox virus, that it might become a worldwide pandemic.	305 (74.3%)	20 (4.8%)	95 (23.1%)
I think that Mpox can add a new burden on the healthcare system of the affected countries.	320 (78%)	40 (9%)	50 (12%)
In my opinion, the Mpox vaccine should be used when available.	230 (56%)	50 (5.4%)	130 (31.7%)
In my opinion, the early detection of the Mpox virus can improve treatment and outcome.	350 (85.3%)	10 (2.4%)	50 (12.1%)
Believe Mpox could become an outbreak in Ethiopia.	90 (21.9%)	150 (36.5%)	170 (41.4%)
Willing to get vaccinated if available	205 (54.8%)	100 (24.3%)	105 (25.6%)
Confidence in the Ethiopian health care system to manage Mpox	120 (29.2%)	42 (10.2%)	248 (60.4%)
Willingness to self‐isolate if exposed	341 (83.1%0	7 (1.7%)	62 (15.1%)
I think that mass media coverage of Mpox may influence its prevention.	345 (84.1)	20 (0.48)	45 (10.9)
I am concerned about the potential spread of mpox within my community/region.	330 (80.4%)	70 (17%)	10 (2%)

Score	Frequency (%)	Remark
< 50	70 (17)	Negative attitude
50–69	30 (7.4)	Fair attitude
≥ 70	310 (75.6)	Positive attitude

Abbreviations: A, agree; D, disagree; N, neutral.

### 3.5 Factors Associated With Medical and Health Science Students' Attitudes to Mpox

In bivariate logistic regression analysis, sex, age, profession, last CGPA, source of information, and sufficient knowledge level were found to be statistically associated with attitude towards mpox (*p* ≤ 0.25). After adjusting this variable using multivariate regression, sex and source of information remained significant predictors of medical and health undergraduate students towards mpox. Hence, the odds of having a positive attitude toward Mpox were 1.583 times higher in female respondents compared to males (AOR = 1.583, 95% CI: (1.024–2.46, *p* = 0.03). Similarly, those getting information by training and from the Websites of the hospital/Ministry of Health 70% (0.16–0.65, *p* = 0.03) 68% (0.16–0.65, *p* = 0.01) more likely to have a positive attitude than those getting information from television, respectively (Table 5).

**Table 5:** Attitude and associated factors of healthcare undergraduate students.

Variables	Attitude		AOR (95% CI)	*p* value
	Positive (%)	Negative (%)		
**Sex**				
Male	117 (28.5)	55 (13.4)	1	
Female	140 (34.1)	98 (23.9)	1.58 (1.024–2.46)	**0.03**
**Age in years**				
18–20	7 (1.7)	2 (0.4)	1	
21–22	161 (39.2)	103 (25.1)	0.40 (0.07–2.14)	0.2
23–25	89 (21.7)	48 (11.7)	0.34 (0.06–1.17)	0.2
**Professions**				
Medicine	68 (16.5)	24 (5.8)	1	
Nurse	86 (20.9)	37 (9)	1.21 (0.57–2.56)	0.61
Midwifery	95 (23.1)	33 (8)	1.23 (0.6–2.54)	0.56
Anesthetist	13 (3.1)	7 (1.7)	1.49 (0.72–3.0)	0.278
Laboratory	36 (8.7)	11 (2.6)	1.09 (0.35–3.3)	0.87
**Information source**				
Television	28 (6.8)	23 (5.6)	1	
Social media	60 (14.6)	59 (14.3)	0.77 (0.3–1.8)	0.55
Websites of the hospital/Ministry of Health	78 (19)	35 (8.5)	0.32 (0.16–0.65)	**0.01**
Friends/family	35 (8.5)	14 (3.4)	0.73 (0.36–1.49)	0.39
Training	61 (14.8)	17 (4.1)	0.3 (0.13–0.67)	**0.03**
**Last CGPA**				
< 2.5	10 (2)	6 (0.1)	1	
2.5–3.74	180 (43.9)	115 (28)	1.28 (0.39–4.1)	0.67
> 3.75	57 (13.9)	42 (10.2)	1.06 (0.61–1.65)	0.98
**Heard Mpox previously**				
Yes	134 (32.6)	75 (18.2)	1.01 (0.65–1.55)	0.96
No	123 (30)	78 (19)	1	
**Knowledge**				
Adequate	95 (23.1)	45 (10.9)	1.13 (0.69–1.85)	0.61
Poor	162 (39.5)	108 (26.3)	1	

*Note: p* < 0.05 (bolded) indicates statistically significant associations.

Abbreviations: AOR, adjusted odds ratio; CI, confidence interval; COR, crude odds ratio.

### 3.6 Practice Towards Mpox

The majority of respondents identified effective preventative strategies for managing mpox, including wearing masks (90.2%), avoiding close contact with people who have suspected or confirmed mpox cases (74.3%), and according to the Ministry of Health's mpox guidelines (72.9%). Over 50% of people take precautions against mpox when traveling (51.4%). Regular hand washing with soap and water or alcohol hand sanitizer was practiced by more than two‐thirds of individuals 51.8% of participants were seen as practicing well (Table 6).

**Table 6:** Practice about Mpox healthcare undergraduate students (*n* = 410).

Questions	Correct answer	Percentage
Wearing a mask when in public or exposed to others (yes)	370	90.2
Washing hands frequently (yes)	350	85.3
If you think you might have Mpox, you can act to protect others by seeking medical advice and isolating from others until you have been evaluated and tested (yes)	211	51.4
Avoiding close contact with someone who has suspected or confirmed Mpox (yes)	305	74.3
If you travel to countries that have Mpox outbreaks, avoid unprotected contact with wild animals, especially those that are sick or dead (including their meat and blood) (yes)	399	97.3
Following the guidelines of the Ministry of Health, if a suspected Mpox(yes)	299	72.9
Following the guidelines of the Ministry of Health, if a suspected Mpox(yes)	250	60.9

Score	Frequency %	Remark
< 50	80 (19.5)	Poor practice
50–69	18 (4.3)	Moderate practice
≥ 70	312 (76.09)	Good practice

### 3.7 Practice and Associated Factors of Mpox

Bivariate logistic regression analysis showed that mpox management practices had a significant association with the sex of the participants, the source of mpox information (social media, hospital, or Ministry of Health websites, friends, family, and training). Participants who had sufficient knowledge and a positive attitude were more likely to have good practices regarding mpox. In multivariate logistic regression analysis, participants who had heard about Mpox before showed higher good practices compared to those who hadn't heard (AOR = 3.72, 95% CI: (2.38–5.80), *p* = 0.002). Also, the study showed that the rate of good practices in those who had sufficient knowledge was 1.07 times greater than that among those who had insufficient knowledge (AOR 1.07, 95% CI: (1.01–2.86), *p* = 0.04). Similarly, participants who had a positive attitude showed higher good practices compared to those who had a negative attitude (AOR = 2.32, 95% CI: (1.44–3.71), *p* = 0.01 (Table 7).

**Table 7:** Practice and associated factors of undergraduate healthcare students.

Variables	Practice		COR (95% CI)	AOR (95% CI)	*p* value
	Good (%)	Poor (%)			
**Sex**					
Male	113 (27.5)	59 (14.3)	1		
Female	141 (34.3)	97 (23.6)	0.75 (0.33–1.50)		0.18
**Age in years**					
> 20	7 (1.7)	2 (0.4)	1		
22–22	157 (38.2)	107 (26)	1,82 (0.36–9.14)		1.77
22–25	90 (21.9)	47 (11.4)	2,38 (0.48–1.04)		1.88
**Professions**					
Medicine	57 (13.9)	35 (8.5)	1		
Nurse	76 (17)	47 (11.4)	1.09 (0.54–2.12)		0.80
Midwifery	77 (18.7)	51 (12.4)	1.16 (0.58–2.31)		
Anesthetist	14 (3.4)	6 (1.4)	1.08 (0.52–2.24)		0.82
Laboratory	30 (7.3)	17 (4.1)	0.75 (0.24–2.33)		0.627
**Information source**					
Television	45 (10.9)	10 (2.4)	1		0.174
Social media	84 (20.4)	31 (7.5)	0.35 (0.15–0.81)		0.01
Websites of the hospital/Ministry of Health	89 (21.7)	35 (8.5)	0.62 (0.33–1.16)		0.136
Friends/family	35 (8.5)	14 (3.4)	8.63 (4.3–17.01)		0.98
Training	41 (10)	26 (6.3)	0.58 (0.30–1.12)		0.25
**Last CGPA**					
< 2.5	11 (2.6)	5 (1.2)	1		
2.5–3.74	179 (43.6)	116 (28.2)	1.18 (0.73–1.93)		0.48
≥ 3.75	64 (15.6)	35 (8.5)	0.83 (0.26–2.58)		0.74
**Heard Mpox previously**					
Yes	169 (41.2)	49 (11.9)	3.19 (1.12–4.30)	3.72 (2.38–5.80)	**0.002**
No	79 (19.2)	122 (29.7)	1		
**Knowledge**					
Adequate	96 (23.4)	44 (10.7)	1.43 (1.28–1.67)	1.07 (1.01–2.86)	**0.04**
Inadequate	132 (32.1)	138 (33.6)	1		
**Attitude**					
Positive	194 (47.3)	97 (23.6)	1.40 (1.26–1.61)	2.32 (1.44–3.71)	**0.01**
Negative	53 (12.9)	66 (16)	1		

*Note: p* < 0.05 (bolded) indicates statistically significant associations

Abbreviations: AOR, Adjusted odds ratio; CI, Confidence interval; COR, Crude odds ratio

## 4 Discussion

This study assessed the knowledge, attitudes, and practices (KAP) toward Mpox among undergraduate medical and health science students. The findings revealed that although most students had heard about Mpox and demonstrated awareness of its basic etiology and transmission routes, significant gaps remained in specific areas of knowledge, particularly regarding vaccination, incubation period, and treatment. Furthermore, information sources and prior awareness of Mpox were significant predictors of knowledge, while sex and information sources were associated with attitudes. In addition, knowledge and attitude levels were important predictors of preventive practices.

In the present study, only 43.9% of students demonstrated good knowledge of Mpox. This finding is consistent with studies conducted among university students in several countries, which also reported limited knowledge regarding Mpox. For example, a study among health science students in Türkiye found that the overall knowledge score was relatively low, indicating important knowledge gaps among future healthcare professionals [21]. Similarly, research among medical students in Saudi Arabia reported that a large proportion of participants had insufficient knowledge regarding Mpox, particularly about symptoms, transmission, and vaccination [22]. These similarities may reflect the relatively recent global emergence of Mpox outbreaks and the limited integration of Mpox‐related content into medical curricula.

Despite the moderate overall knowledge level, many participants in this study correctly identified that Mpox is caused by a virus and recognized major transmission routes such as contact with infected individuals, animals, and contaminated body fluids. This level of awareness may be attributed to the extensive global media coverage and public health messaging during the 2022–2024 Mpox outbreaks. However, substantial knowledge gaps were identified regarding the availability of vaccines and the protective role of the smallpox vaccine. Similar misconceptions were reported in previous studies where many participants were unaware of the cross‐protective effect of smallpox vaccination against Mpox infection [22]. Such gaps highlight the need for improved education about emerging infectious diseases in health science training programs.

The present study also showed that 75.6% of participants had a positive attitude toward Mpox prevention. This result aligns with findings from studies conducted among university students in other settings, where positive attitudes toward preventive measures were relatively common despite limited knowledge levels. For instance, research among university students reported that a majority of participants expressed concern about the disease and supported preventive actions to control its spread [23]. Positive attitudes may reflect the perceived seriousness of emerging infectious diseases following the global experience with the COVID‐19 pandemic.

However, confidence in the healthcare system's preparedness to manage Mpox was relatively low among respondents in this study. This finding may reflect broader concerns about the readiness of healthcare systems in resource‐limited settings to respond effectively to emerging infectious diseases. Global assessments have shown that many low‐ and middle‐income countries still experience significant gaps in epidemic preparedness, including limited detection, response, and operational capacities [24]. In addition, evaluations of health system preparedness for infectious disease outbreaks in middle‐income and low‐resource contexts have highlighted weaknesses in infrastructure, logistics, and workforce capacity that can undermine effective outbreak management [25]. Similar challenges were observed across healthcare facilities in sub‐Saharan Africa during the COVID‐19 pandemic, where a lack of essential supplies and preparedness structures hindered timely responses to emergent threats [26].

Regarding preventive practices, 51.8% of participants demonstrated good practices, including mask use, avoiding close contact with infected individuals, and adherence to Ministry of Health guidelines. These findings are comparable to previous studies, indicating that preventive behaviors are often influenced by both knowledge and attitudes toward infectious diseases. Evidence suggests that individuals with better knowledge and more positive attitudes are more likely to adopt protective behaviors and preventive health practices [27].

In this study, the source of information was a significant predictor of knowledge and attitudes, with students who received information through training, social media, and official health websites being more likely to have better knowledge. Similar findings have been reported in other studies where information obtained from health institutions and official websites was associated with higher levels of awareness and understanding of Mpox [28]. This highlights the importance of reliable information sources in shaping accurate knowledge and appropriate perceptions about emerging diseases.

Additionally, this study found that female students were more likely to demonstrate positive attitudes toward Mpox compared with male students. This finding is consistent with previous research indicating that female students often exhibit greater health awareness and more positive health‐related attitudes. For example, a multicenter Mpox study reported higher levels of knowledge and awareness among female health science students compared with males, suggesting gender differences in engagement with public health information [28]. Similarly, research among medical and pharmacy students in Vietnam found that female students had better Mpox knowledge and attitudes compared with male counterparts [29]. Moreover, studies on medical students' attitudes toward psychological help‐seeking also showed that female students had more positive attitudes than male students, reflecting a broader pattern of gender differences in health attitudes [30].

Finally, the results indicated that students with sufficient knowledge and positive attitudes were more likely to demonstrate good preventive practices. This relationship between knowledge, attitudes, and practices has been consistently reported in previous studies and supports the theoretical framework that improved knowledge can positively influence attitudes and behavioral outcomes. A systematic review of Mpox KAP studies found that higher knowledge and positive attitudes were significantly associated with better preventive practices among participants [31]. Similarly, research in infectious disease contexts, including COVID‐19 and Mpox, has shown that knowledge and attitudes predict engagement with preventive health behaviors [32, 33].

### 4.1 Strength of the Study

The study's strongest point is that it is the first to evaluate Ethiopian undergraduate healthcare students' knowledge, attitudes, and concerns about mpox. Consequently, the findings of this study could be useful in modifying relevant educational and awareness initiatives and courses that aim to improve comprehension of virus emergence. This could improve future healthcare students' health‐related behavior.

### 4.2 Limitations of the Study

This study was conducted at a single location; its results might not be representative of other areas or medical facilities with distinct sociodemographics. The use of self‐reported data may induce response bias tendencies, which could compromise the reported knowledge and attitudes, and the correctness of practice scores. Furthermore, the cross‐sectional study design limits the ability to infer a causal relationship between the identified factors and outcomes. In spite of these drawbacks, this study will serve as a crucial foundation for upcoming studies and initiatives on Mpox management and prevention in comparable environments.

## 5 Conclusion and Recommendation

### 5.1 Conclusion

This study found that undergraduate healthcare students had suboptimal knowledge of Mpox, with nearly half demonstrating poor knowledge despite adequate awareness of its cause, transmission, and common symptoms. Important gaps were identified regarding vaccination, incubation period, and treatment. Nevertheless, students exhibited a generally positive attitude and reported good preventive practices. Prior awareness, adequate knowledge, and positive attitude were significantly associated with better practice, indicating that improved education can enhance preparedness for emerging infectious diseases.

### 5.2 Recommendations

Integrating emerging infectious diseases into undergraduate curricula, providing regular training and awareness programs, and promoting the use of reliable institutional information sources are recommended to address knowledge gaps. Strengthening educational interventions and preparedness activities may further improve students' competence and confidence in responding to future outbreaks.

## Author Contributions

**Esubalew Muluneh Aligaz:** conceptualization, investigation, methodology, data curation. **Zekarias Markos:** investigation, writing – original draft, validation, visualization, writing – review and editing. **Fikadu Tadesse Diress:** methodology, formal analysis, resources. **Sitotaw Tesfa Zegeye:** supervision.

## Funding

The authors have nothing to report.

## Ethics Statement

The Declaration of Helsinki and the Institutional Research Ethics Board's guidance were adhered to in the present study. Under the Declaration of Helsinki, which sets moral standards for medical research involving human subjects, the study was carried out. The University of Wachamo College of Medicine's Ethical Review Committee gave its approval to this study (Ref: WCU‐0300/2025). All participants gave their electronic informed consent before starting the questions. All participants received information about the study's objectives and advantages on the first page of the questionnaire, along with guarantees of information privacy and confidentiality.

## Conflicts of Interest

The authors declared no conflicts of interest.

## Transparency Statement

The lead author Esubalew Muluneh Aligaz affirms that this manuscript is an honest, accurate, and transparent account of the study being reported; that no important aspects of the study have been omitted; and that any discrepancies from the study as planned (and, if relevant, registered) have been explained.

## Supporting information

Supporting File

## Data Availability

The datasets used and analyzed during the current study are available from the corresponding authors upon reasonable request.
