# Supplementary material for: Assessment of Knowledge, Attitudes, and Practices Towards Monkeypox and Associated Factors Among Undergraduate Healthcare Students in Ethiopia, 2025: A Cross‐Sectional Study
Source: Health Sci Rep. 2026 May 11;9(5):e72513. doi: 10.1002/hsr2.72513 (PMC13161470; doi:10.1002/hsr2.72513)
Supplement: Supplementary file 1 — Supporting File [file HSR2-9-e72513-s001.docx]

# Supplementary Material 1: Data Collection Questionnaire

Title: Knowledge, Attitude, and Practice Toward Mpox Among Undergraduate Healthcare Students

Instructions to Participants:
You are invited to participate in this study assessing knowledge, attitudes, and practices related to Mpox. Your responses are confidential and will be used only for research purposes. Please answer all questions honestly.

## Section I: Socio-Demographic Characteristics

1. Age (in years): ______

2. Sex: ☐ Male ☐ Female

3. Marital Status: ☐ Single ☐ Married ☐ Other

4. Field of Study: ☐ Medicine ☐ Nursing ☐ Midwifery ☐ Anesthesia ☐ Medical Laboratory

5. Last CGPA: ______

6. Have you ever heard about Mpox? ☐ Yes ☐ No

7. Source of Information about Mpox (Select all that apply): ☐ Social media ☐ Television/Radio ☐ Classroom lectures ☐ scientific articles ☐ Health professionals’ ☐ other: ______________

8. Have you received formal training about Mpox?
☐ Yes
☐ No

## Section II: Knowledge about Mpox (Yes / No / I Don’t Know)

9. Mpox is a viral disease.

10. Mpox can be transmitted from animals to humans.

11. Mpox can spread through close physical contact with an infected person.

12. Skin rash is a common symptom of Mpox.

13. Fever is an early sign of Mpox infection.

14. Mpox can be transmitted through contaminated materials (e.g., bedding, clothing).

15. Proper hand hygiene helps prevent Mpox transmission.

16. Isolation of infected individuals helps control the spread of Mpox.

17. Vaccination can provide protection against Mpox.

18. Mpox is usually self-limiting but may cause complications.

19. Healthcare workers are at higher risk of exposure to Mpox.

## Section III: Attitude Toward Mpox (Agree / Neutral / Disagree)

20. Mpox is a serious public health concern.

21. I feel at risk of being exposed to Mpox during clinical practice.

22. Infection prevention measures can effectively control Mpox spread.

23. I am willing to follow isolation guidelines if exposed to Mpox.

24. I believe healthcare students should receive training on Mpox prevention.

25. Using personal protective equipment (PPE) is essential when managing suspected cases.

26. Public awareness is important to prevent Mpox outbreaks.

27. Ethiopia’s healthcare system can effectively manage a Mpox outbreak.

## Section IV: Practice Related to Mpox Prevention (Yes / No / Sometimes)

28. I regularly practice proper hand hygiene in clinical settings.

29. I use personal protective equipment when required during patient care.

30. I follow infection prevention guidelines during clinical attachments.

31. I actively seek information about emerging infectious diseases like Mpox.

32. I educate peers or patients about infection prevention methods.

33. I avoid direct contact with suspected infectious lesions without protection.

34. I report suspected infectious cases according to clinical protocol
